# Long-term health-related quality of life and mental health in patients with immune thrombotic thrombocytopenic purpura

**DOI:** 10.1007/s00277-024-05771-3

**Published:** 2024-04-27

**Authors:** Olga Mulas, Fabio Efficace, Alessandro Costa, Thomas Baldi, Filippo Zerbini, Daniela Mantovani, Emanuela Morelli, Daniela Perra, Giorgio La Nasa, Giovanni Caocci

**Affiliations:** 1 SC Ematologia e CTMO, Ospedale Businco, ARNAS Brotzu, Cagliari, Italy; 2 Dipartimento di Scienze Mediche e Sanità Pubblica, Università di Cagliari, Cagliari, Italy; 3 Health Outcomes Research Unit, Italian Group for Adult Hematologic Diseases (GIMEMA) Data Center, Rome, 00161 Italy

**Keywords:** TTP, HRQoL, Fatigue, FACT-Cog, HADS

## Abstract

Immune thrombotic thrombocytopenic purpura (iTTP) is a rare and potentially life-threatening disorder. Treatment advances have lowered morbidity rates, but past acute events can still cause long-term consequences, reducing health-related quality of life (HRQoL) and determining cognitive impairment, anxiety, and depression. We aimed to investigate these aspects and the role of caplacizumab and rituximab: 39 patients were evaluated using the Medical Outcomes Study 36-Item Short Form Health Survey (SF-36), the FACIT-Fatigue, the Hospital Anxiety and Depression Scale, and the Functional Assessment in Cancer Therapy-Cognitive Function questionnaires. The median age at study inclusion was 50 years (IQR 38–60), and the median follow-up from diagnosis was 97 months (IQR 14–182); 82% of patients were female, and 36% had one or more recurrences. Caplacizumab was administered in 16 patients (41%), as well as rituximab. ITTP patients reported lower physical and mental HRQoL scores than the general population. No differences in physical or mental domains were observed between patients treated or not with caplacizumab, while those who received rituximab reported lower scores in mental health. Neurological impairment at diagnosis correlated with worse fatigue. The majority of patients (72%) reported anxiety or depression (82%). ITTP had a significant impact on the long-term cognitive function, fatigue, depression, and anxiety levels of patients, with a negative effect on their HRQoL. Our findings underscore the need to pay special attention to patients’ long-term physical and mental health, regardless of the medical treatments received.

**Supplementary Information:**

The online version contains supplementary material available at 10.1007/s00277-024-05771-3.

## Introduction

Immune Thrombotic Thrombocytopenic Purpura (iTTP) is a rare and severe disorder that can be life-threatening and recurring. It results from a deficiency of ADAMTS13, a von Willebrand factor (vWF)-cleaving protease that leads to microvascular thrombosis in various organs [1]. To manage iTTP effectively, accurate diagnosis and comprehensive care from a team of specialists trained in managing bleeding disorders are crucial, along with access to ongoing treatment [2].

The cornerstone of life-saving therapy in iTTP has been therapeutic plasma exchange (TPE) combined with immunomodulatory strategies. Caplacizumab was approved for treating iTTP in August 2018 after favorable results from the TITAN and HERCULES clinical trials [3, 4]. It is a nanobody that inhibits the interaction between ultra-large von Willebrand factor multimers and platelets [5]. Patients who received caplacizumab in addition to standard care showed a significantly shorter time to platelet count normalization, a reduction in duration of TPE, and a reduction of days of hospitalization compared to those who received a placebo [3, 4]. However, high rates of relapses may occur, and refractory disease with fatal outcomes still occurs [6]. In this context, rituximab, a B-cell-depleting therapy, has represented the second breakthrough in iTTP management [7]. Recently, rituximab has been proposed as part of a first-line strategy for patients with unfavorable outcomes under standard care [8].

While these treatments have been successful, it’s essential to consider the long-term complications of iTTP [9, 10] Survivors of iTTP may experience neurological issues and depression as a common mental long-term complication, which can lead to a decrease in health-related quality of life (HRQoL) and reduced resilience. All these aspects, including cognitive impairment, anxiety, and depression disorders, are equally essential to be considered in managing patients with iTTP, both evaluating the long-term implications of previous acute episodes and providing psychological support to those who need it [2]. Although previous efforts have addressed these factors, limited understanding remains regarding the impact of new therapeutic approaches on HRQoL and mental health.

The primary objective of this study was to compare the long-term HRQoL of iTTP patients with that of the general population. Secondary objectives were to evaluate how the addition of caplacizumab and rituximab to standard treatment affects HRQoL domains and explore the impact of depression and anxiety on treatment outcomes. Additionally, a cognitive evaluation was conducted.

## Materials and methods

### Study design and data collection

Eligible patients for this cross-sectional study were recruited from those diagnosed with iTTP in the Hematology Department of Businco Hospital in Cagliari, Italy. All patients were initially contacted by telephone or mail to inform them of the study’s purposes. Survey instruments and standardized HRQoL patient questionnaires were submitted to patients through a dedicated website. Enrollment began in July 2022 and ended in July 2023. Sociodemographic and clinical information data were retrieved from hospital medical records. The study was performed in accordance with the Declaration of Helsinki and was approved by the Ethics Committee of Cagliari (authorization number: PROT. PG/2021/11,283). All patients provided written informed consent.

### Generic HRQoL and fatigue assessment

The Medical Outcomes Study 36-Item Short-Form Health Survey (SF-36) was used to assess HRQoL [11, 12]. This questionnaire was translated and adapted to the cultural context in Italy as part of the International Quality of Life Assessment project [13]. The SF-36 (version 1) is a questionnaire consisting of 36 items that aim to evaluate a patient’s self-reported health and functioning. This tool examines eight domains of HRQoL: physical functioning (PF), role limitations due to physical functioning (RP), bodily pain (BP), general health (GH), vitality (VT), social functioning (SF), role emotional functioning (RE), and mental health (MH). Based on these eight scales, two higher-order component scores, the physical component score (PCS) and mental component score (MCS), are calculated using a weighted combination of the eight scales [14, 15].

Fatigue was assessed with the Functional Assessment of Chronic Illness Therapy–Fatigue Scale (FACIT-Fatigue), a self-report questionnaire consisting of 13 items. Each item is rated on a five-point Likert scale, with a total score ranging from 0 (representing the worst fatigue) to 52 (indicating no fatigue). Scores ≤ 30 were considered indicative of clinically significant fatigue, as per the guidelines of the scale creators, based on data from the general population [16].

### Anxiety, depression, and cognitive assessment

The Hospital Anxiety and Depression Scale (HADS) and the Functional Assessment in Cancer Therapy–Cognitive Function (FACT-Cog) were used to assess anxiety and depression and cognitive aspects. The HADS questionnaire is a widely used tool for measuring anxiety and depression levels in patients with physical illness. It consists of 14 items, with seven items indicating anxiety and the other seven indicating depression. Each item has four response options, scored with values ranging from 0 to 3, resulting in a scale value between 0 and 21 for each scale. The original test authors defined three ranges for each scale: 0–7 (non-cases), 8–10 (doubtful cases), and 11–21 (cases). The HADS total score can be calculated by adding up the anxiety and depression items [17].

The FACT-Cog is a self-reported questionnaire comprising 37 items assessing cognitive function in the past seven days. The questionnaire provides four domain scores: perceived cognitive impairments (PCI), impact of perceived cognitive impairments on quality of life (QoL), comments from others (Oth), and perceived cognitive abilities (PCA). The scores vary for each scale because they are calculated by adding up the FACT-Cog item scores. Higher scores indicate better cognitive functioning [18, 19].

### Statistical analysis

Differences in mean scores and corresponding 95% confidence intervals (CI) of SF-36 scales between iTTP patients and the general population were graphically displayed. To account for possible confounders in HRQoL outcomes, samples were previously matched by age and sex. Namely, a 1:1 nearest neighbor optimal matching was implemented, using the difference among propensity scores as the distance measure. Post-matching balance of variables was checked on both matching and adjusting variables. Means differences were also adjusted by age and sex. Furthermore, observations in either the iTTP sample or normative data were discarded with missing values in at least one matching or adjusting variable. Overlap condition of propensity scores in the case and potential control group was ensured before matching, discarding observations outside the standard range of propensity scores. An 8-point difference on the eight SF-36 scales was considered a minimal important difference (MID), whereas a difference of 2 points was judged as a MID for the PCS and MCS scores. A score difference at least equal to a MID was considered to represent a clinically meaningful difference [14, 20].

Mean scores of the FACIT Fatigue, HADS, and FACT-Cog scales were calculated. A multivariable linear regression analysis was also performed to examine the association with the following variables: sex, age, comorbidities (autoimmune, neurological), laboratory values (hemoglobin and platelets levels), numbers of Previous iTTP relapse before survey, treatments (number of TPE, use of caplacizumab, use of rituximab), time to response, follow-up time, episode free survival (EFS), time lower or higher than 12 months between iTTP and survey.

Mean scores of SF-36 scales were also stratified by neurological comorbidities, number of previous iTTP relapses before the survey, use of caplacizumab, use of rituximab and time lower or higher than 12 months between iTTP and survey; possible differences between groups were assessed using Wilcoxon-Mann-Whitney test.

All statistical tests were two-sided with type I error α = 0.05. Because of the study’s exploratory nature, we did not adjust for multiple testing. The association of sociodemographic and clinical variables with patients reported outcomes (PRO) scores was investigated by Wilcoxon and Kruskall-Wallis tests. The Pearson correlation coefficient was used to assess the strength and direction of the linear relationship between PRO scores. An absolute correlation coefficient value (IRI) of 0.70 and above indicates a strong correlation, a moderate correlation between 0.40 and 0.70, and a weak correlation for values below 0.4 [21].

## Results

We evaluated, between October 2022 and August 2023, a group of 82 patients diagnosed with iTTP at our institution between 2002 and 2023 to determine their eligibility. Out of the total, 43 patients were excluded from the study. The reasons for exclusion were as follows: 10 patients were misdiagnosed and did not have iTTP, 22 patients were lost during the follow-up period, and 11 patients did not complete the consent form. A total of 39 patients were finally included in the study. Their clinical characteristics are presented in Table 1. At diagnosis, 10 (26%) of patients reported autoimmune comorbidities, and 5 (13%) had neurological impairment. The median age at study inclusion was 50 years (IQR: 38–60), and the median follow-up from diagnosis was 97 months (IQR: 14–182). Fourteen (36%) patients had experienced at least one iTTP relapse before the study enrollment. The median time between the last episode of iTTP and the survey was 41 months (IQR: 6–77). Twelve (31%) patients experienced iTTP recurrence or the first episode within 12 months before the survey. The median number of TPE sessions was 7 (IQR: 7–9), and the hematological response median time was 7 days (IQR: 5–10). Caplacizumab was administered in 16 patients (41%) with a median dosing duration of 28 days (IQR: 19–30). Rituximab was incorporated into the therapeutic regimen of 16 patients (41%), with 7 (18%) of them receiving it as their initial treatment. The results of PRO are reported in supplemental Table 1.

**Table 1:** Clinical characteristics of 39 iTTP patients

At diagnosis	
Sex female, N (%)	32 (82)
Age at diagnosis, median years (IQR)	40 (28–51)
Autoimmune comorbidities, N (%)	10 (26)
Neurological comorbidities, N (%)	5 (13)
Median follow-up, median months (IQR)	97 (14–182)

Parameters at last iTTP event before survey	
Hemoglobin g/dl, median value (IQR)	8.3 (7–9.6)
Platelet ×10^3^/uL, median value (IQR)	12 (9–20)
Previous iTTP relapse, N (%)	14 (36)
TPE, median (IQR)	7 (5–9)
Caplacizumab association, N (%)	16 (41)
Caplacizumab dosing, median days (IQR)	28 (19–30)
Rituximab association, N (%)	16 (41)
Rituximab first line, N (%)	7 (18)
Time of hematological response, median days (IQR)	7 (5–10)
Age at survey, median years (IQR)	50 (38–60)
Time between last iTTP event and survey, median months (IQR)	41 (6–77)
Patients with lower than 12 months between iTTP and survey, N (%)	12 (31)

TPE: therapeutic plasma exchange; IQR = interquartile range

### Comparison of HRQoL profile of iTTP patients with the general population

**Fig. 1:**
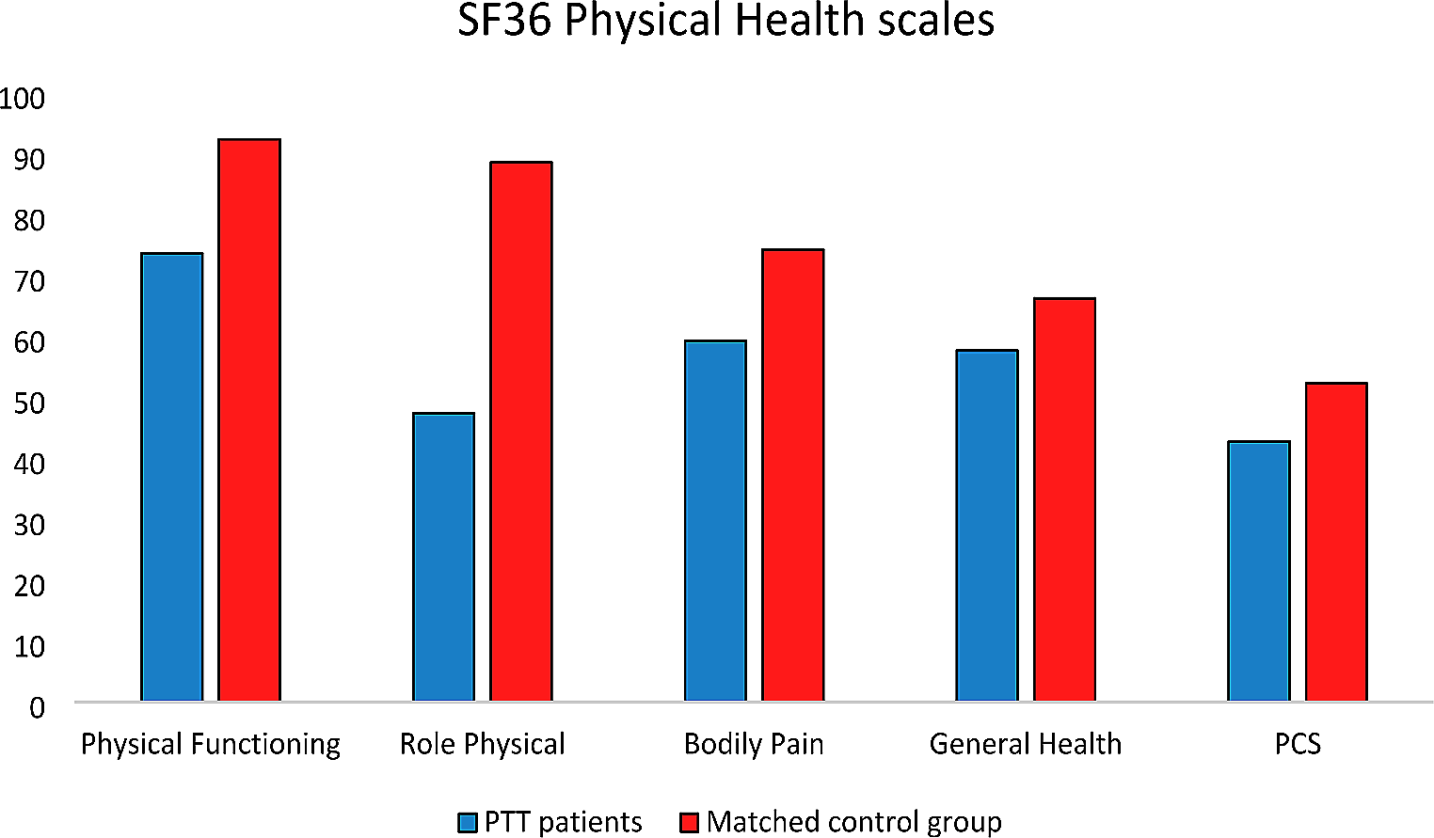
Physical health reported by the SF36 questionnaire PCS: physical component summary; SD: standard deviation; CI: confidence interval * Exceeds clinically important difference (i.e. 8 points for the SF-36 scales and 2 points for the PCS scores); + Statistically significant

**Fig. 2:**
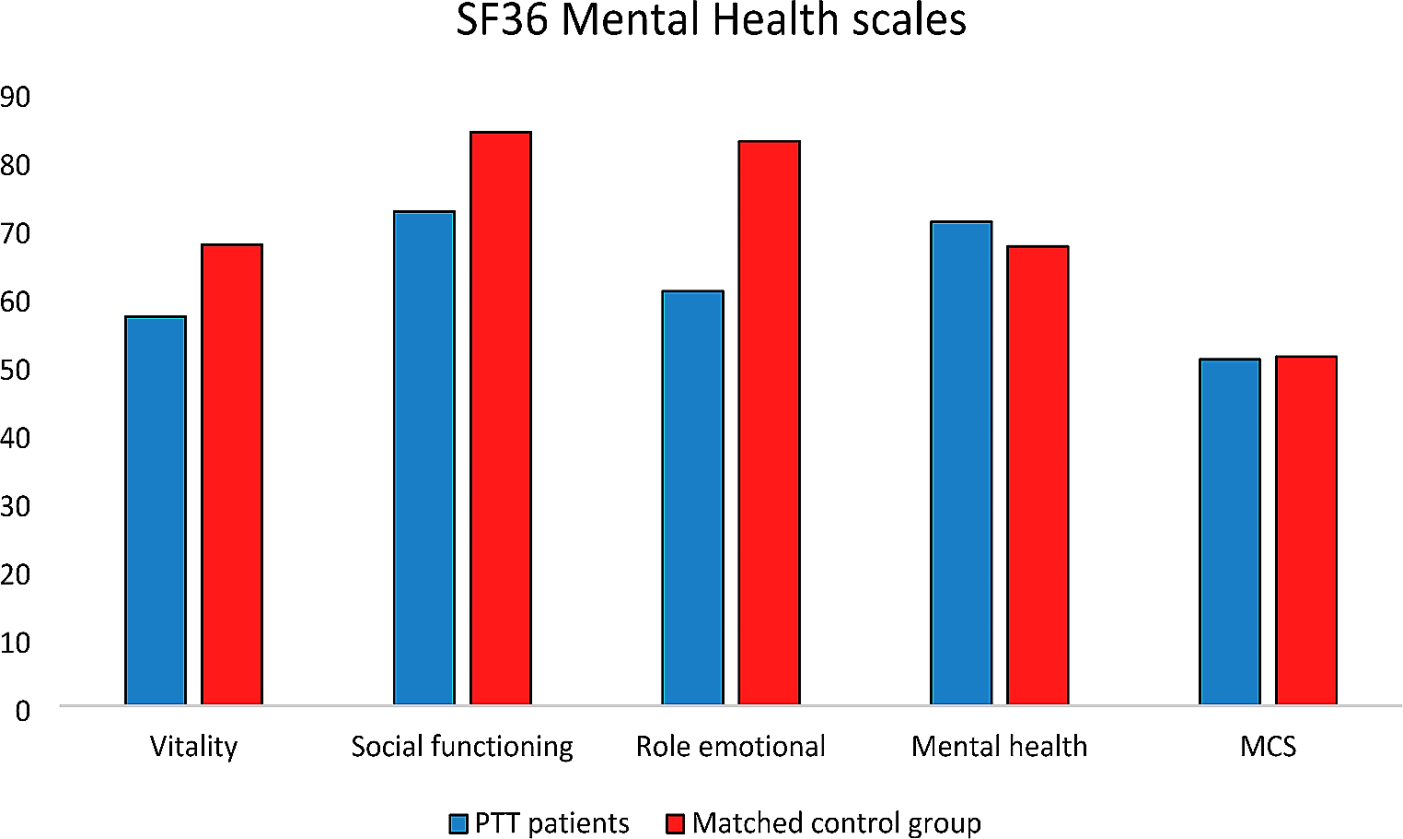
Mental health reported by the SF36 questionnaire MCS: mental component summary; SD: standard deviation; CI: confidence interval * Exceeds clinically important difference (i.e. 8 points for the SF-36 scales and 2 points for the MCS scores); + Statistically significant

About physical health-related domains, statistically and clinically meaningful differences were observed in PF (Δ=-18.7; 95%CI: -26.97, -10.35; *p* < 0.0001), RP (Δ=-41.2; 95%CI: -54.27, -28.07; *p* < 0.0001), and BP scale (Δ=-15; 95%CI: -25.32, -4.74; *p* = 0.02) (Fig. 1). Regarding mental health-related domains, statistically and clinically meaningful differences were found in VT (Δ=-10.5; 95%CI: -19.10, -1.82; *p* = 0.04), SF (Δ=-11.6; 95%CI: -20.85, -2.29; *p* = 0.04) and RE scale (Δ=-22; 95%CI: -36.35, -7.57; *p* = 0.01) (Fig. 2). Adjusted mean score differences for the PCS and MCS were Δ=-9.5; 95%CI: -12.30, -6.68, *p* < 0.01 and Δ=-0.4; 95%CI: -4.62, 3.86, *p* = 0.88 respectively.

### Exploratory analysis of HRQoL by clinical and treatment characteristics

No significant differences were found in physical and mental components between patients treated with caplacizumab and those who had not received it ( Table 2). In addition, the group of patients with more than 12 months elapsed between iTTP episode and the current survey had a better profile in the GH (63 ± 19 vs. 46 ± 20, *p* = 0.027) and SF scale (79 ± 27 vs. 57 ± 27, *p* = 0.025).

**Table 2:** SF36 questionnaire by clinical variables, with relative *p*-value for mean differences

	Neuro comorbidities			Relapse number			Caplacizumab			Rituximab			EFS Class		
SF36	No *N* = 34^1^	Yes *N* = 5^1^	p^2^	0 *N* = 25^1^	≥ 1 *N* = 14^1^	p^2^	No, *N* = 23^1^	Yes, *N* = 16^1^	p^2^	No *N* = 32^1^	Yes *N* = 7^1^	p^2^	< 12 months *N* = 12^1^	≥ 12 months *N* = 27^1^	p^2^
PF	77 (20)	52 (46)	0.4	74 (24)	73 (29)	0.8	73 (24)	75 (29)	0.3	75 (25)	66 (31)	0.4	69 (35)	76 (20)	0.7
RP	52 (41)	15 (22)	0.06	42 (42)	57 (37)	0.3	57 (41)	34 (38)	0.10	47 (41)	50 (41)	0.9	35 (41)	53 (40)	0.2
BP	60 (31)	57 (40)	0.8	56 (30)	65 (36)	0.4	60 (32)	59 (32)	0.9	57 (32)	70 (32)	0.3	59 (33)	59 (32)	0.9
GH	58 (20)	55 (28)	0.8	61 (19)	53 (22)	0.3	62 (20)	52 (20)	0.2	57 (20)	61 (23)	0.7	46 (20)	63 (19)	0.02
PCS	44 (9)	38 (9)	0.2	42 (9)	45 (8)	0.3	44 (9)	41 (8)	0.5	42 (9)	46 (9)	0.3	41 (10)	44 (8)	0.5
VT	60 (25)	37 (34)	0.2	59 (27)	54 (27)	0.6	60 (29)	53 (24)	0.4	59 (25)	49 (36)	0.6	54 (28)	58 (27)	0.6
SF	76 (27)	50 (35)	0.10	72 (28)	74 (31)	0.7	76 (27)	68 (31)	0.5	75 (27)	63 (35)	0.4	57 (28)	79 (27)	0.02
RE	64 (44)	40 (43)	0.2	64 (43)	55 (48)	0.6	67 (45)	52 (44)	0.3	67 (42)	33 (47)	0.09	44 (46)	68 (43)	0.13
MH	74 (23)	51 (34)	0.08	75 (23)	63 (28)	0.12	71 (28)	71 (21)	0.8	75 (21)	54 (36)	0.15	67 (22)	72 (27)	0.3
MCS	52 (13)	42 (14)	0.2	52 (13)	48 (13)	0.3	52 (14)	49 (12)	0.5	53 (12)	42 (15)	0.06	47 (11)	52 (14)	0.11

^1^Mean (SD)

^2^Wilcoxon rank sum test; Wilcoxon rank sum exact test

EFS: episode free survival; PF: physical functioning; RP: role limitations due to physical functioning; BP: bodily pain; GH: general health; VT: Vitality; SF: social functioning; RE: role emotional functioning; MH: mental health; PCS: physical component score; MCS: mental component score

### Factors associated with physical and mental health-related aspects

Older age was associated with lower PCS scores (*p* = 0.04). Factors associated with higher scores on the MCS scale were younger (*p* = 0.04), male sex (*p* = 0.04), absence of autoimmune comorbidities (*p* = 0.05), and no treatment with rituximab (*p* = 0.03) (Supplemental Tables 2 and 3).

### Fatigue by clinical characteristics

The FACIT-Fatigue Scale mean score of the total sample was 36.7 ± 13. Neurological comorbidity was associated with lower significant scores compared with patients without neurological impairment at diagnosis (20.2 ± 18.1 and 39.2 ± 10.4, *p* = 0.001, respectively). No differences were observed based on the presence of iTTP recurrences (36.8 ± 13.3 and 36.7 ± 13.2, *p* = 0.98, respectively), the time of survey collection before or after 12 months the last iTTP episode (33.9 ± 13.7 and 38 ± 12.8, *p* = 0.37, respectively), the use of caplacizumab (35.1 ± 11.9 and 37.9 ± 13.9, *p* = 0.51 respectively) or rituximab (33 ± 17.7 and 37.6 ± 12, *p* = 0.4 respectively) (supplemental Table 4). In multivariate analysis, a significant correlation was found between less fatigue and male sex (𝜷=10.2, 95%CI: 0.8, 19.6: *p* = 0.03) and the absence of neurological comorbidities at diagnosis (𝜷=16.9, 95%CI: 6.1, 27.7: *p* < 0.01), (Supplemental Tables 4 and 5).

### Anxiety, depression, and cognitive functioning

The HADS score for the entire sample was 21.4 ± 3.1. The anxiety scale showed that 28 out of 39 patients (72%) reported a clear state of anxiety, while 8 (20%) were classified as doubtful cases. On administering the depression scale, it was found that approximately 82% of the patients showed a borderline (51%) condition or abnormal (31%) results. The scores for anxiety and depression were 11.9 ± 3 and 9.6 ± 2, respectively, as depicted in supplemental Table 1.

In thirty-eight patients, the FACT-Cog was collected. The means ± SD of PCI, QoL, Oth, and PCA were 55 ± 17.3, 11.5 ± 4.9, 14.8 ± 2.2 and 16.2 ± 7.6 respectively. A significant correlation in univariate analysis was found with better FACT-Cog scores and absence of autoimmune (𝜷=17.2, 95%CI: 5.49, 28.9: *p* = 0.005) or neurological comorbidities at diagnosis (𝜷=25.3, 95%CI: 10.5, 40.1: *p* = 0.001). Multivariate analysis also confirmed the first association (𝜷=12.7, 95%CI: -0.02, 25.4: *p* = 0.05) (supplemental Table 6).

**Fig. 3:**
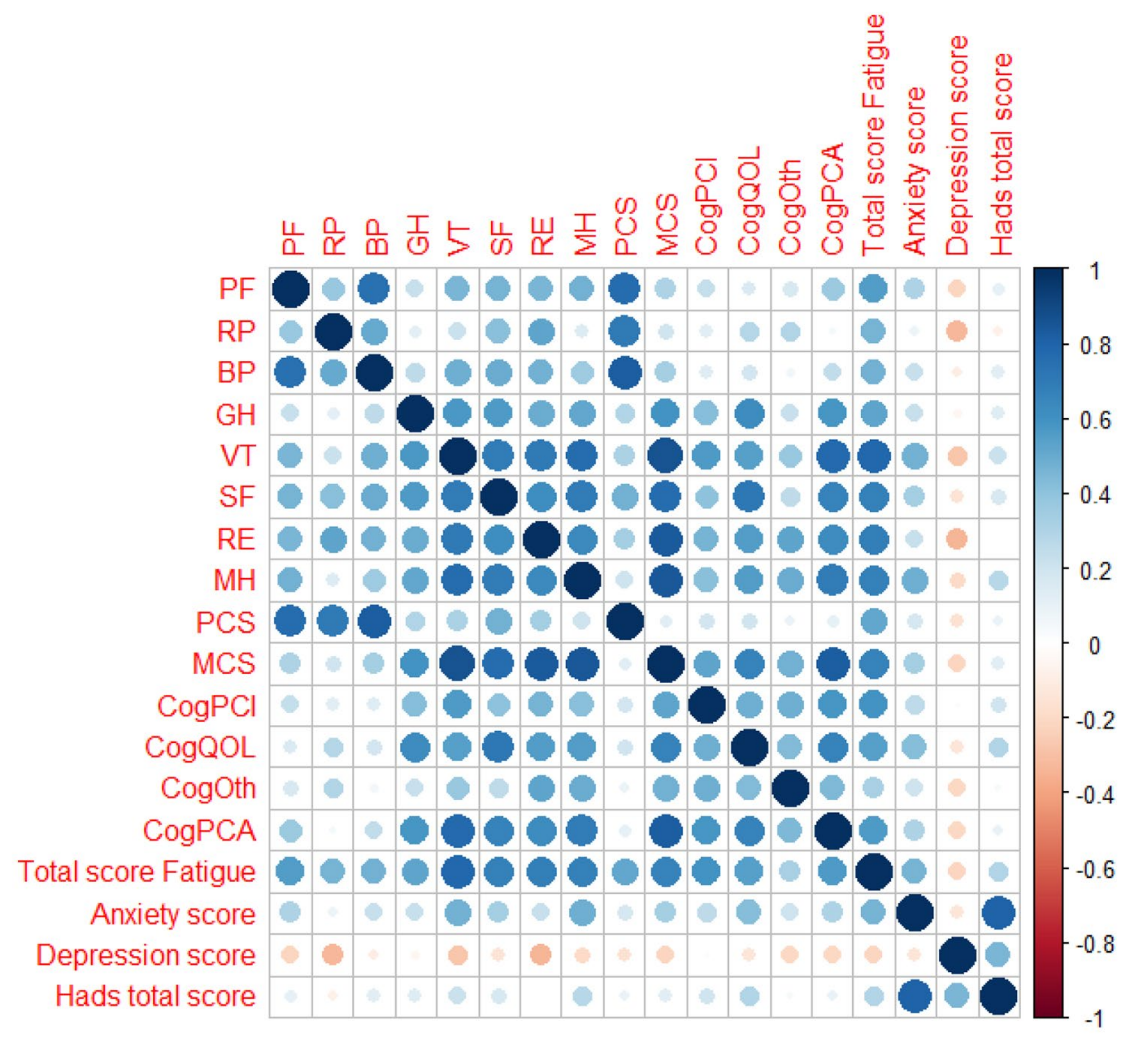
Pearson’s correlation matrix between physical and mental items of SF36, Facit-fatigue, HADS, and FACT-Cog questionnaires PF: physical functioning; RP: role limitations due to physical functioning; BP: bodily pain; GH: general health; VT: Vitality; SF: social functioning; RE: role emotional functioning; MH: mental health; PCS: physical component score; MCS: mental component score; CogPCI: perceived cognitive impairments; CogQOL: impact of perceived cognitive impairments on quality of life; CogOth: comments from others; CogPCA: perceived cognitive abilities; A correlation matrix is a tool used to measure the relationship between two variables in a dataset. The matrix is a table where each cell displays a correlation coefficient that ranges between − 1 and 1. It also uses different colors and circle sizes to visually display the correlation, with stronger relationships indicated by bigger circles and darker colors

The correlation matrix indicated a significant association between the FACIT-Fatigue and SF36 scores and all Fact-Cog items, with r coefficients greater than 0.4. High levels of FACT-Cog correlated with better HRQoL scores, particularly in the MCS (Fig. 3 and supplemental Table 7).

## Discussion

Advancements in therapeutic approaches have significantly improved patients’ prognoses with iTTP, enabling them to achieve long-term survival rates [7, 22]. However, survivors frequently experience chronic problems that can have a remarkable effect on the psychological and cognitive well-being of the individual [23].

Very few studies have reported data on long-term HRQoL and the mental status of iTTP patients [2, 24–29]. As the first objective, the present study indicates that the HRQoL profile after a median follow-up of eight years from the iTTP occurrence is lower than that of the general population. This finding is consistent with previous studies that have reported a high prevalence of depression and anxiety, a more negative attitude toward life, and low resilience among iTTP patients. Resilience was also found to be negatively correlated with the severity of the depression [2].

While it is true that advancements in iTTP treatment have reduced hospitalizations and improved hematological recovery, the survivors are at increased risk of multiple adverse health outcomes, including higher than all-cause mortality rates [10]. In fact, according to the Oklahoma iTTP registry, iTTP patients may experience comorbidities such as an increase in body mass index, hypertension, and other autoimmune conditions like systemic lupus erythematosus, as well as depression [9]. Studies have also suggested that iTTP patients with ischemic brain lesions detected by magnetic resonance imaging may experience cognitive impairment [27, 30]. Our study revealed that patients without any neurological or autoimmune conditions generally had enhanced HRQoL and lower levels of fatigue, indicating that the early detection and treatment of underlying conditions that may have adverse effects on their cognitive and psychological health is crucial.

In our secondary endpoint, we found that the vast majority of our patients reported anxiety, depression, and impaired mental profiles, regardless of the type of treatment received. Although the post-HERCULES study did not provide conclusive findings on the long-term effects of iTTP on HRQoL and cognitive function [31], we found that patients who received caplacizumab treatment did not experience stable or slightly improved cognitive functioning and HRQoL. While rituximab may reduce the risk of iTTP relapse [7, 32], our study found that it did not lead to higher HRQoL scores, even when used as a first-line treatment. Surprisingly, patients who did not receive rituximab reported better mental profiles. This aligns with similar observations in using rituximab for immune thrombocythemia, where there was no clear link between the drug’s response and improved HRQoL [33]. The lower scores observed in patients treated with rituximab may be due to the drug being reserved for more severe presentations or relapses of the disease, which could inevitably affect their quality of life.

A web-based survey tool, consisting of demographic and clinical data and two validated self-administered questionnaires, was utilized to gather information from a cohort of 236 TTP patients to estimate the prevalence of symptoms related to post-traumatic stress disorder (PTSD) and depression in iTTP survivors [34]. PTSD is an adverse reaction to traumatic experiences that results in patients repeatedly reliving the traumatic event (such as through recurring thoughts or nightmares), avoiding anything that reminds them of the event, and experiencing hyperarousal symptoms (like irritability and difficulty sleeping), which cause adverse effects on their cognitive ability and functioning. All of these factors contribute to a lower quality of life. The study revealed a high incidence of PTSD and depression in iTTP survivors. This is because patients who were previously in good health have experienced a traumatic, life-threatening event and now face an uncertain future, including the risk of recurrence [34]. Our findings confirm that a majority of patients exhibit significant symptoms of anxiety and depression based on their responses to the HADS questionnaires, highlighting the need for psycho-educational strategies [25].

Additionally, as reported in Fig. 2, the mental component scores in the areas of VT, SF, and RE were lower than those of the general population, indicating the need for further support and interventions to improve the patient’s mental health. A study by Riva and colleagues involved 35 patients with acquired iTTP. The objective was to evaluate the long-term neuropsychological effects of the condition at a minimum of three months after their last acute iTTP episode [25]. The neuropsychological assessment results indicated below-average direct, indirect, and deferred memory scores. Despite receiving plasma exchange and immunosuppressive therapy during the acute phase of iTTP, patients continue to experience long-term neurological complications and impaired HRQoL even years after the acute phase [25]. Lewis et al. confirmed that persistent cognitive problems and impaired HRQoL are common among iTTP survivors and suggested that defining these mental abnormalities could help develop targeted rehabilitation techniques. This, in turn, would enable better adaptation and emotional support for patients, addressing their concerns about feeling neglected due to the lack of acknowledgment and treatment for their health problems [24]. In our study, we utilized the FACT-Cog questionnaire to analyze cognitive impairment, and our findings indicate a strong correlation between the score obtained in the questionnaire and HRQoL, particularly in the mental domain. The results in Fig. 3 demonstrate a strong relationship between the various areas examined by FACT-Cog and the different aspects analyzed by SF36, particularly those related to mental health. Using simple questionnaires to assess cognitive status, clinicians could identify patients requiring further evaluation. Combining these tools could further enhance these patients’ quality of care and well-being.

Although our study provides valuable information, it has some limitations. First, the number of patients enrolled in a single institution was small. Second, the patients included in the study had different stages of the disease, such as diagnosis, relapse, or follow-up, which could result in bias. However, considering the rarity of this disease, the data we have gathered still hold significant importance. They could help facilitate more targeted supportive care interventions.

In conclusion, our research shows that although cutting-edge drugs have greatly improved the course of iTTP, they still report HRQoL impairments compared to their peers in the general population. We collected an average follow-up of almost 8 years and used multidimensional PRO that were closely related to each other. It seems that anxiety and depression were commonly observed, along with a more widespread impairment of mental and cognitive functions.

We used FACT-Cog for the first time in iTTP, which showed significant agreement with other questionnaires. The findings indicate that FACT-Cog could help evaluate the cognitive function of patients with iTTP. It is an effective method for assessing the cognitive sphere in these individuals. The sustained presence of impairments during long-term follow-up highlights the necessity for consistent monitoring and support for those affected by the disease.

Finally, it appears essential to rephrase the treatment approach for these patients. The new approach should focus on engaging multiple specialists, such as hematologists, neurologists, psychiatrists, psychologists, and rehab experts, in managing the patients. This multitasking approach should consider not only the patient’s physical health but also their psychological well-being to ensure psychophysical rehabilitation.

### Electronic supplementary material

Below is the link to the electronic supplementary material.

Supplementary Material 1

## Data Availability

No datasets were generated or analysed during the current study.
