# Supplementary material for: Long-term health-related quality of life and mental health in patients with immune thrombotic thrombocytopenic purpura
Source: Ann Hematol. 2024 Apr 27;103(7):2523–31. doi: 10.1007/s00277-024-05771-3 (PMC11224107; doi:10.1007/s00277-024-05771-3)
Supplement: Supplementary file 1 — Supplementary Material 1 [file 277_2024_5771_MOESM1_ESM.docx]

| **Measure and Items** | **n** | **mean** | **SD** | **Range** |
| --- | --- | --- | --- | --- |
| **SF36** |  |  |  |  |
| *Physical health* |  |  |  |  |
| Physical functioning | 39 | 73.7 | 25.7 | 0-100 |
| Role physical | 39 | 47.4 | 40.5 | 0-100 |
| Bodily pain | 39 | 59.3 | 31.8 | 0-100 |
| General health | 39 | 57.7 | 20.5 | 0-87 |
| PCS | 39 | 42.8 | 8.7 | 24.8-57 |
| *Mental health* |  |  |  |  |
| Vitality | 39 | 57 | 26.7 | 0-100 |
| Social functioning | 39 | 72.4 | 28.7 | 0-100 |
| Role emotional | 39 | 60.7 | 44.5 | 0-100 |
| Mental health | 39 | 70.9 | 25.4 | 0-100 |
| MCS | 39 | 50.7 | 13.1 | 22.5-72.3 |
| **FACIT-Fatigue** | 39 | 36.7 | 13 | 1-50 |
| **HADS** | 39 | 21.4 | 3.1 | 14-27 |
| Anxiety | 39 | 11.9 | 3 | 5-18 |
| Depression | 39 | 9.6 | 2 | 7-14 |
| **FACT-Cog** |  |  |  |  |
| PCI | 38 | 55 | 17.3 | 9-72 |
| QoL | 38 | 11.5 | 4.9 | 0-16 |
| Oth | 38 | 14.8 | 2.2 | 6-16 |
| PCA | 38 | 16.2 | 7.6 | 1-28 |

**Supplemental table 1. Patient scores reported on the PRO questionnaires.**

CogPCI, perceived cognitive impairments; CogQOL, impact of perceived cognitive impairments on quality of life; CogOth, comments from others; CogPCA, perceived cognitive abilities

| **SF36-PCS** | **Univariate Analysis** | | **Multivariate Analysis** | |
| --- | --- | --- | --- | --- |
| **Variables** | **β (95% C.I.)** | **p-value** | **β (95% C.I.)** | **p-value** |
| *Age at diagnosis (years)* | -0.22 (-0.43; -0.01) | **0.04** | -0.22 (-0.43; -0.01) | **0.04** |
| *Sex (Male vs Female)* | 6.11 (-1.09;13.33) | 0.09 | NA | NA |
| *Autoimmune comorbidities (Absent vs Present)* | 5.12 (-1.24;11.48) | 0.11 | NA | NA |
| *Neurological comorbidities (Absent vs Present)* | 5.54 (-2.85;13.95) | 0.19 | NA | NA |
| *Onset with neurological symptomatology* | 3.08 (-2.58;8.75) | 0.28 | NA | NA |
| *Number of relapses before questionnaire (0 vs ≥ 1)* | -2.9 (-8.89;2.93) | 0.31 | NA | NA |
| *Haemoglobin level (g/dl)* | -0.74 (-2.33;0.844) | 0.34 | NA | NA |
| *PLT count (mm^3^)* | 0.01435 (-0.15291;0.018162) | 0.86 | NA | NA |
| *Number of plasmapheresis sessions* | 0.09 (-0.38;0.55) | 0.71 | NA | NA |
| *Received caplacizumab (No vs Yes)* | 2.33 (-3.46;8.13) | 0.42 | NA | NA |
| *Number of caplacizumab doses* | -0.13 (-0.36;0.08) | 0.21 | NA | NA |
| *Received rituximab (No vs Yes)* | -3.47 (-9.2;2.25) | 0.23 | NA | NA |
| *Received rituximab in first line (No vs Yes)* | -4.43 (-11.78;2.92) | 0.23 | NA | NA |
| *Response time (days)* | 0.15 (-0.18;0.48) | 0.36 | NA | NA |
| *Follow-up time (months)* | -0.002 (-0.03;0.023) | 0.89 | NA | NA |
| *EFS class (<12 vs >=12)* | -2.97 (-9.13;3.18) | 0.33 | NA | NA |

**Supplemental table 2.** **Univariate and Multivariate Analysis with SF36-PCS as outcome**

| **SF36-MCS** | **Univariate Analysis** | | **Multivariate Analysis** | |
| --- | --- | --- | --- | --- |
| **Variables** | **β (95% C.I.)** | **p-value** | **β (95% C.I.)** | **p-value** |
| *Age at diagnosis (years)* | 0.15 (-0.18;0.49) | 0.35 | 0.34 (0.01;0.66) | **0.04** |
| *Sex (Male vs Female)* | 7.62 (-3.34;18.58) | 0.17 | 11.41 (0.64;22.18) | **0.04** |
| *Autoimmune comorbidities (Absent vs Present)* | 6.05 (-3.63;15.73) | 0.21 | 15.24 (5.15;25.32) | **0.004** |
| *Neurological comorbidities (Absent vs Present)* | 9.72 (-2.78;22.22) | 0.12 | NA | NA |
| *Onset with neurological symptomatology* | -3.14 (-11.7;5.43) | 0.46 | NA | NA |
| *Number of relapses before questionnaire (0 vs ≥ 1)* | 4.85 (-4;13.7) | 0.27 | NA | NA |
| *Haemoglobin level (g/dl)* | -0.94 (-3.33;1.45) | 0.43 | NA | NA |
| *PLT count (mm^3^)* | 0.02682(-0.22423;0.27786) | 0.83 | 0.17231 (-.06328; 0.40790) | 0.15 |
| *Number of plasmapheresis sessions* | -0.27 (-0.97;0.42) | 0.44 | NA | NA |
| *Received caplacizumab (No vs Yes)* | 2.51 (-6.22;11.25) | 0.56 | NA | NA |
| *Number of caplacizumab doses* | -0.07 (-0.41;0.26) | 0.66 | NA | NA |
| *Received rituximab (No vs Yes)* | 9.67(1.50;17.83) | **0.02** | 8.81 (1;16.6) | **0.03** |
| *Received rituximab in first line (No vs Yes)* | 10.92(0.27;21.57) | **0.05** | NA | NA |
| *Response time (days)* | -0.10(-0.61;0.39) | 0.67 | NA | NA |
| *Follow-up time (months)* | 0.02 (-0.01;0.06) | 0.27 | NA | NA |
| *EFS class (<12 vs >=12)* | -5.56(-14.72;3.61) | 0.23 | NA | NA |

**Supplemental table 3.** **Univariate and Multivariate Analysis with SF36-MCS as outcome**

| **Total score** | **Categories** | **Number** | **Mean (SD)** | **Median (IQR)** | **Range** | **p** |
| --- | --- | --- | --- | --- | --- | --- |
| **Overall** | Overall |  | 36.7 (13) | 42 (32 – 46) | 1 - 50 |  |
| **Neuro comorb** | Present | 5 | 20.2 (18.1) | 17 (6-33) | 1 - 44 | 0.0014 |
|  | Absent | 34 | 39.2(10.4) | 42 (35-46) | 3 - 50 |  |
| **Recidive number** | 0 | 25 | 36.7(13.2) | 42 (35-44) | 3 - 50 | 0.98 |
|  | >=1 | 14 | 36.8 (13.3) | 41 (30-46) | 1 - 50 |  |
| **Caplacizumab** | Yes | 16 | 35.1 (11.9) | 38 (31-43.5) | 6 - 49 | 0.51 |
|  | No | 23 | 37.9 (13.9) | 42 (35-48) | 1 - 50 |  |
| **Rituximab** | Yes | 7 | 33 (17.7) | 39 (17-48) | 1 - 50 | 0.40 |
|  | No | 32 | 37.6 (12) | 42 (32.5-45) | 3 - 50 |  |
| **EFS class** | <12 | 12 | 33.9 (13.7) | 38 (25.5-45.5) | 6 - 49 | 0.37 |
|  | >=12 | 27 | 38 (12.8) | 42 (35-46) | 1 - 50 |  |

**Supplemental table 4.** **Facit-Fatigue total score, by clinical variables, with relative p-value for mean differences.**

EFS, episode free survival

| **FACIT Fatigue** | **Univariate Analysis** | | **Multivariate Analysis** | |
| --- | --- | --- | --- | --- |
| **Variables** | **β (95% C.I.)** | **p-value** | **β (95% C.I.)** | **p-value** |
| *Age at diagnosis (years)* | -0.16 (-0.49;0.17) | 0.32 | NA | NA |
| *Sex (Male vs Female)* | 12.8 (2.5;23.2) | **0.02** | 10.2 (0.8;19.6) | **0.03** |
| *Autoimmune comorbidities (Absent vs Present)* | 7.45 (-2.04;16.9) | 0.12 | NA | NA |
| *Neurological comorbidities (Absent vs Present)* | 18.98 (7.8;30.1) | **0.001** | 16.9 (6.1;27.7)) | **0.003** |
| *Onset with neurological symptomatology* | -2.5 (-11;6.1) | 0.56 | NA | NA |
| *Number of relapses before questionnaire (0 vs ≥ 1)* | -0.2 (-9.1;8.8) | 0.97 | NA | NA |
| *Haemoglobin level (g/dl)* | -1.1 (-3.4;1.3) | 0-37 | NA | NA |
| *PLT count (mm^3^)* | 0.04493 ( -0.20408;0.29394) | 0.72 | NA | NA |
| *Number of plasmapheresis sessions* | -0.27 (-0.96;0.4) | 0.43 | NA | NA |
| *Received caplacizumab (No vs Yes)* | 2.7 (-5.9;11.4) | 0.52 | NA | NA |
| *Number of caplacizumab doses* | -0-05 (-0.39;0.28) | 0.74 | NA | NA |
| *Received rituximab (No vs Yes)* | 2.32 (-6.4;11) | 0.59 | NA | NA |
| *Received rituximab in first line (No vs Yes)* | 4.56 (-6.5;15.6) | 0.4 | NA | NA |
| *Response time (days)* | 0.02 (-0.5;0.5) | 0.92 | NA | NA |
| *Follow-up time (months)* | 0.02 (-0.01;0.06) | 0.17 | NA | NA |
| *EFS time (months)* | 0.01 (-0.02;0.06) | 0.37 | NA | NA |

**Supplemental table 5.** **Univariate and Multivariate Analysis with FACIT-Fatigue as outcome**

| **FACIT Cognitive** | **Univariate Analysis** | | **Multivariate Analysis** | |
| --- | --- | --- | --- | --- |
| *Variables* | *β (95% C.I.)* | *p-value* | *β (95% C.I.)* | *p-value* |
| *Age at diagnosis (years)* | *-0.27 (-0.72;0.18)* | *0.23* | *NA* | *NA* |
| *Sex (Male vs Female)* | *13.6 (-0.5;27.8)* | *0.06* | *11.2 (-2.2;24.7)* | *0.09* |
| *Autoimmune comorbidities (Absent vs Present)* | *17.2 (5.49;28.9)* | *0.005* | *12.7 (-0.02;25.4)* | *0.05* |
| *Neurological comorbidities (Absent vs Present)* | *25.3 (10.5;40.1)* | *0.0013* | *14.6 (-1.6;30.9)* | *0.08* |
| *Onset with neurological symptomatology* | *-3.59 (-15.1;7.9)* | *0.53* | *NA* | *NA* |
| *Number of relapses before questionnaire (0 vs ≥ 1)* | *0.34 (-11.6;12.3)* | *0.95* | *NA* | *NA* |
| *Haemoglobin level (g/dl)* | *-3.1 (-6.2; -0.09)* | *0.04* | *NA* | *NA* |
| *PLT count (mm3)* | *-0.19086 ( -0.51674;0.13502)* | *0.24* | *NA* | *NA* |
| *Number of plasmapheresis sessions* | *0.1 (-0.8;1.03)* | *0.81* | *NA* | *NA* |
| *Received caplacizumab (No vs Yes)* | *3.4 (-8.3;15.2)* | *0.56* | *NA* | *NA* |
| *Number of caplacizumab doses* | *-0.18 (-0.6;0.3)* | *0.43* | *NA* | *NA* |
| *Received rituximab (No vs Yes)* | *-0.21 (-11.9;11.5)* | *0.97* | *NA* | *NA* |
| *Received rituximab in first line (No vs Yes)* | *7.7 (-6.9;22.4)* | *0.29* | *NA* | *NA* |
| *Response time (days)* | *0.22 (-0.4;0.9)* | *0.49* | *NA* | *NA* |
| *Follow-up time (months)* | *0.02 (-0.03;0.07)* | *0.36* | *NA* | *NA* |
| *EFS time (months)* | *0.02 (-0.02;0.08)* | *0.36* | *NA* | *NA* |

**Supplemental table 6. Univariate and Multivariate Analysis with FACT-Cognitive as outcome**

|  | **PF** | **RP** | **BP** | **GH** | **VT** | **SF** | **RE** | **MH** | **PCS** | **MCS** | **CogPCI** | **CogQOL** | **CogOth** | **CogPCA** | **Total.score.Fatigue** | **Anxiety.score** | **Depression.score** | **Hads.total.score** |
| --- | --- | --- | --- | --- | --- | --- | --- | --- | --- | --- | --- | --- | --- | --- | --- | --- | --- | --- |
| PF | 1 | 0,376153 | 0,744436 | 0,2252 | 0,450303 | 0,463512 | 0,45496 | 0,470506 | 0,762859 | 0,309838 | 0,235849 | 0,159802 | 0,169428 | 0,361603 | 0,552744 | 0,302898 | -0,21674 | 0,103929 |
| RP | 0,376153 | 1 | 0,501133 | 0,116743 | 0,210211 | 0,416366 | 0,521172 | 0,146408 | 0,707185 | 0,198929 | 0,126994 | 0,289471 | 0,292508 | 0,043732 | 0,466174 | 0,07376 | -0,32792 | -0,07756 |
| BP | 0,744436 | 0,501133 | 1 | 0,252101 | 0,482881 | 0,493988 | 0,472968 | 0,356535 | 0,827901 | 0,333157 | 0,137992 | 0,18684 | 0,058685 | 0,240904 | 0,472266 | 0,228987 | -0,09278 | 0,129705 |
| GH | 0,2252 | 0,116743 | 0,252101 | 1 | 0,579352 | 0,566613 | 0,490227 | 0,513504 | 0,29265 | 0,591798 | 0,424363 | 0,622364 | 0,223292 | 0,58415 | 0,52668 | 0,221985 | -0,0493 | 0,139942 |
| VT | 0,450303 | 0,210211 | 0,482881 | 0,579352 | 1 | 0,696829 | 0,709436 | 0,766738 | 0,313434 | 0,863848 | 0,565964 | 0,546231 | 0,376458 | 0,772111 | 0,788923 | 0,477664 | -0,27259 | 0,21621 |
| SF | 0,463512 | 0,416366 | 0,493988 | 0,566613 | 0,696829 | 1 | 0,614707 | 0,690437 | 0,47503 | 0,764901 | 0,402375 | 0,716658 | 0,253135 | 0,667367 | 0,676372 | 0,339582 | -0,14737 | 0,169613 |
| RE | 0,45496 | 0,521172 | 0,472968 | 0,490227 | 0,709436 | 0,614707 | 1 | 0,630858 | 0,334147 | 0,834209 | 0,468772 | 0,55117 | 0,522967 | 0,629052 | 0,680996 | 0,228371 | -0,33557 | -0,00912 |
| MH | 0,470506 | 0,146408 | 0,356535 | 0,513504 | 0,766738 | 0,690437 | 0,630858 | 1 | 0,207742 | 0,844784 | 0,410589 | 0,558436 | 0,493965 | 0,693396 | 0,679305 | 0,487574 | -0,19942 | 0,275647 |
| PCS | 0,762859 | 0,707185 | 0,827901 | 0,29265 | 0,313434 | 0,47503 | 0,334147 | 0,207742 | 1 | 0,121255 | 0,183751 | 0,196827 | 0,081706 | 0,103519 | 0,518076 | 0,17958 | -0,15202 | 0,0815 |
| MCS | 0,309838 | 0,198929 | 0,333157 | 0,591798 | 0,863848 | 0,764901 | 0,834209 | 0,844784 | 0,121255 | 1 | 0,521668 | 0,66429 | 0,475063 | 0,822772 | 0,661564 | 0,338471 | -0,21972 | 0,129747 |
| **CogPCI** | 0,235849 | 0,126994 | 0,137992 | 0,424363 | 0,565964 | 0,402375 | 0,468772 | 0,410589 | 0,183751 | 0,521668 | 1 | 0,483838 | 0,480508 | 0,586293 | 0,596728 | 0,258767 | -0,0267 | 0,199098 |
| **CogQOL** | 0,159802 | 0,289471 | 0,18684 | 0,622364 | 0,546231 | 0,716658 | 0,55117 | 0,558436 | 0,196827 | 0,66429 | 0,483838 | 1 | 0,430436 | 0,660835 | 0,545902 | 0,423376 | -0,13951 | 0,295408 |
| **CogOth** | 0,169428 | 0,292508 | 0,058685 | 0,223292 | 0,376458 | 0,253135 | 0,522967 | 0,493965 | 0,081706 | 0,475063 | 0,480508 | 0,430436 | 1 | 0,447533 | 0,326116 | 0,203922 | -0,20328 | 0,036452 |
| **CogPCA** | 0,361603 | 0,043732 | 0,240904 | 0,58415 | 0,772111 | 0,667367 | 0,629052 | 0,693396 | 0,103519 | 0,822772 | 0,586293 | 0,660835 | 0,447533 | 1 | 0,563745 | 0,302441 | -0,20592 | 0,093709 |
| **Total.score.Fatigue** | 0,552744 | 0,466174 | 0,472266 | 0,52668 | 0,788923 | 0,676372 | 0,680996 | 0,679305 | 0,518076 | 0,661564 | 0,596728 | 0,545902 | 0,326116 | 0,563745 | 1 | 0,468837 | -0,2133 | 0,290844 |
| **Anxiety.score** | 0,302898 | 0,07376 | 0,228987 | 0,221985 | 0,477664 | 0,339582 | 0,228371 | 0,487574 | 0,17958 | 0,338471 | 0,258767 | 0,423376 | 0,203922 | 0,302441 | 0,468837 | 1 | -0,13082 | 0,800226 |
| **Depression.score** | -0,21674 | -0,32792 | -0,09278 | -0,0493 | -0,27259 | -0,14737 | -0,33557 | -0,19942 | -0,15202 | -0,21972 | -0,0267 | -0,13951 | -0,20328 | -0,20592 | -0,2133 | -0,13082 | 1 | 0,456047 |
| **Hads.total.score** | 0,103929 | -0,07756 | 0,129705 | 0,139942 | 0,21621 | 0,169613 | -0,00912 | 0,275647 | 0,0815 | 0,129747 | 0,199098 | 0,295408 | 0,036452 | 0,093709 | 0,290844 | 0,800226 | 0,456047 | 1 |

**Supplemental table 7.** **Pearson’s correlation matrix.**

PF, physical functioning; RP, role limitations due to physical functioning; BP, bodily pain; GH, general health; VT, Vitality; SF, social functioning; RE, role emotional functioning; MH, mental health; PCS, physical component score; MCS, mental component score; CogPCI, perceived cognitive impairments; CogQOL, impact of perceived cognitive impairments on quality of life; CogOth, comments from others; CogPCA, perceived cognitive abilities
